# HAT2 mediates histone H4K4 acetylation and affects micrococcal nuclease sensitivity of chromatin in *Leishmania donovani*

**DOI:** 10.1371/journal.pone.0177372

**Published:** 2017-05-09

**Authors:** Pravin K. Jha, Mohd. Imran Khan, Anshul Mishra, Pradeep Das, Kislay K. Sinha

**Affiliations:** 1 Department of Biotechnology, National Institute of Pharmaceutical Education and Research, Hajipur, Bihar, India; 2 Molecular Biology Division, Rajendra Memorial Research Institute of Medical Sciences, Patna, Bihar, India; Ludwig-Maximilians-Universitaet Muenchen, GERMANY

## Abstract

Histone post-translational modifications (PTMs) such as acetylation and methylation are known to affect chromatin higher order structures. Primary targets of these modifications include basic residues present at N-terminus tail region of core histones. Four histone acetyltransferase (HAT) genes have been identified in trypanosomatids. HAT1, HAT3 and HAT4 of *Leishmania donovani* have been partially characterized. However, there is no report about HAT2 of *Leishmania donovani*. Lysine residues present on the N-terminal tail of *Leishmania donovani* histone H4 are conserved in other trypanosomatids and humans. PTMs of lysines modulate various functions at chromatin level. The four histone acetyltransferases encoded in Leishmania genome were over-expressed to analyse their functional activity. All four HATs were found actively acetylating core histones H3/H4. Similar to *L*. *donovani* HAT3 and HAT4, HAT2 was found to be a member of MYST family protein and have SAS2 type domain. Over-expression of HAT2 significantly increases acetylation of H4K4. To analyse the effect of HAT2 over-expression on chromatin accessibility, micrococcal nuclease digestion assay was performed. MNase digestion resulted in a higher proportion of the mononucleosomes and dinucleosomes in HAT2 over-expressing cells as compared to WT *L*. *donovani* cells. Acetylation of lysine-4 neutralizes the amino terminal region of histone H4. This weakens its interaction with neighbouring nucleosomes and the linker DNA. HAT2 over-expression in *L*. *donovani* resulted in highly accessible chromatin suggesting chromatin decondensation. HAT2 may have an important role to play in global regulation of transcription in *L*. *donovani*. Better understanding of these epigenetic determinants of parasite would help in designing novel therapeutic strategies.

## Introduction

Histones are highly conserved across the eukaryotes and play crucial role in packaging of genome. Octamer of core histones wrapped around by 146 bp of DNA constituting nucleosomes which further condense to form chromatin higher order structures [1]. Highly condensed structure is called heterochromatin whereas the less dense form is euchromatin [2]. The genes in euchromatin are more likely to be transcriptionally active. The packing of chromatin is affected by many factors including the incorporation of histone variants, chromatin remodelling complexes and post translational modifications (PTMs) of core histone proteins viz. H2A, H2B, H3 and H4 [3–6]. N-terminal tail region of histones protruding out of nucleosome core along with C-terminal of histone H2A are major sites for PTMs. Although these histone marks are thought to be more prone to PTMs affecting histone-DNA and inter-nucleosomal interactions, previous studies suggest that the core domain modifications also affect chromatin conformation [7]. Recently, the role of histone PTM was implicated in two life stages of *T*. *cruzi* i.e. epimastigote and trypomastigote [8]. Out of several PTMs, acetylation and methylation most commonly regulate transcriptional activity at chromatin level. Several families of histone modifying enzymes have been characterised including Histone Acetyltransferases (HATs), Histone Deacetylases (HDACs) and Histone Methyltransferases (HMTs) [9–16].

Acetylation of histone N-terminal tail enriched with basic amino acids such as lysine and arginine by different histone acetyltransferases is among the most studied histone modification occurring in chromatin. At physiological pH, these basic amino acids are positively charged, which neutralizes the negative charge of phosphate backbone of DNA thus helping in packaging of DNA. Changes in acetylation level of lys/arg residues present on histone tails modulate its interaction with DNA and therefore, overall compactness of chromatin. This compactness affects various DNA transactions such as chromosome assembly [17], replication [18], transcription [19], recombination [20], repair [21], etc. and provides a fundamental basis for various regulations at chromatin level. Hyper-acetylation on core histone proteins makes chromatin more relaxed and transcriptionally active [22, 23]. The decondensation of chromatin fibre has been commonly assayed by DNase hypersensitivity in various studies [24].

Trypanosomatids comprising *Trypanosoma cruzi*, *T*. *brucei* and *Leishmania spp*. are among the earliest branching eukaryotes. Phylogenetic analysis based on protein sequence comparison suggests that human histones are closer to that of yeast than to that of trypanosomatids. Sequence comparison particularly in amino terminus of different species ascertains trypanosomal histones as most divergent histones [25, 26]. Previous studies suggest that the types and locations of PTMs on N- terminal histone tails are highly conserved among trypanosomes. da Cunha et al. have demonstrated that histones H3 and H2B of *T*. *cruzi* are mostly methylated while histone H4 and H2A are predominantly acetylated. Mass spectrometric analysis of H4 indicated acetylation at positions 4, 10, 14 and 57. Out of these, lysine 4 is acetylated in majority of histone H4, while other acetylations at the N-terminus portion of histone H4 are less abundant [27]. This was supported by studies in *T*. *brucei* where histone H4 remains ~80% acetylated at lysine 4 [28, 29]. A lower level of acetylation was detected for lysine 10 (~6%) and lysine 2 and 3 (~2%) [29]. However, acetylation at lysine 10 and 14 is also required for chromatin remodelling needed for transcription and replication [30]. PTMs in H4 N-terminus of *T*. *brucei* are unusually very high as compared to other organisms [31].

PTMs of specific residues at N-terminal of core histones in *L*. *donovani* have gained significant importance in recent time. *L*. *donovani* HAT3 has been functionally characterised and demonstrated to modulate DNA repair as well as cell cycle events [32]. However, HAT4 acetylates N-terminus of Leishmania histone H4; it doesn’t affect acetylation of H2A, H2B and H3. The primary target of HAT4-mediated acetylation is the H4K14. Also, acetylation of the C-terminus of H2A has not been reported in Leishmania [33]. In *L*. *major*, the H to H (head to head/divergent) strand switch regions (SSRs) which are sites for polycistronic transcriptional initiation are found to have higher levels of histone acetylation indicating higher accessibility of transcription machinery to these regions [34].

Transcription at chromatin level is regulated by compactness of the chromatin in turn changing the accessibility of RNA polymerase and transcription factors to genes and particularly, promoters. PTMs by various enzymes have been shown to directly affect the relaxation and compaction of chromatin. The present study reports the role of *Leishmania donovani* HAT2 in acetylation of core histone H4 and its effect on chromatin digestion by micrococcal nucleases. This further provides an indication that HAT2 is associated with nucleosomal compaction.

## Materials and methods

### Leishmania culture

*Leishmania donovani* Ag83 promastigotes were cultured in M199 media with Hanks salt (Gibco) at pH 7.4 as described previously [35]. It was supplemented with 10% FBS (Gibco) and 100U/ml penicillin streptomycin (Gibco) as described previously. It was incubated at 25°C in a BOD incubator.

### Genomic DNA isolation

Culture containing 1x10^9^ cells of *L*. *donovani* promastigotes was centrifuged at 4500g and genomic DNA was isolated using DNA isolation kit (Qiagen). DNA was eluted in 200 μl DNase free double distilled water and quantified using Nanodrop (Thermo Fisher Scientific).

### Cloning of *Leishmania donovani* HAT2

HAT2 gene was cloned for over-expression in *L*. *donovani*. Clone was prepared using pLPneo2 vector (kindly gifted by Greg Matlashewski, McGill University). Gene was amplified using *L*. *donovani* genomic DNA as template and gene specific primers; HAT2_F: TTTTAAGCTTACAATGGCAGTCGCGCAGTCCGC and HAT2_R: TTTTCTCGAGTCAGCGTGTGCTGGTGTGAG. The amplified PCR product was purified and digested with HindIII and XhoI restriction enzymes. Similarly, pLPneo2 vector was also digested with HindIII and XhoI. Both vector and insert were gel purified and ligated using T4 DNA ligase (Fermentas). The ligated product was transformed into *E*. *coli* DH5α and transformants were selected on ampicillin containing LB agar plate. Clones were confirmed by colony PCR and release of insert using double digestion with HindIII and XhoI.

### Transfection of cloned HAT2

Clones were transfected by electroporation as described previously by Robinson and Beverley [36]. Briefly, 1 x 10^8^ mid-log phase cells (per transfection) were pelleted at 1000*g* for 10 minutes and resuspended in half the original volume in ice cold Cytomix buffer (120 mM KCl, 0.15 mM CaCl_2_, 10 mM K_2_HPO_4_, 25 mM HEPES, 2 mM EDTA, 2 mM MgCl_2_; pH 7.6). Cells were pelleted again and resuspended in ice cold Cytomix buffer to a final concentration of 2 x 10^8^ cells/ml. 8–10 μg of pLPneo2-HAT2 or pLPneo2 plasmids were aliquoted into 4 mm gap cuvettes (Bio-Rad) and 500 μl of cells were added to each cuvette and mixed by pipetting. Electroporation was done twice at 25 μF, 1500 V (3.75 kV/cm) with a gap of 10 seconds between pulses (Gene Pulser Xcell Electroporator, Bio-Rad). The electroporated cells were incubated on ice for 10 minutes and transferred in a flask with 10 ml M199 medium supplemented with 20% FBS. The flask was incubated at 25°C. After 24 hours, media was replaced with an additional supplementation of 100 μg/ml G418 (HiMedia) for selection of over-expressing cells.

### Histone isolation

Histones were isolated from WT, Vector (pLPneo2) transfected and HAT2 over-expressing *L*. *donovani* cultures. Isolation was performed using Histone extraction kit (Abcam). Briefly, cells were harvested by centrifugation at 1000g for 5 minutes at 4°C and resuspended (10^7^ cells/ml) in pre-lysis buffer. Cells were lysed by incubating on ice for 10 minutes with gentle stirring. It was centrifuged at 8,000g for 1 minute at 4°C and the pellet was resuspended in lysis buffer (200 μl/10^7^cells). It was incubated on ice for 30 minutes and centrifuged at 11,000g for 5 minutes at 4°C. The supernatant containing acid soluble proteins were transferred to a new vial and 0.3 volume of Balance-DTT buffer was added and mixed. The protein was quantified using BCA protein assay kit (Pierce).

### Micrococcal nuclease treatment assay

MNase assay was performed using protocol described earlier with slight modification suitable for *Leishmania donovani* [37]. 2.5 ml of log phase cells (2x10^7^ cells/ml) were centrifuged and resuspended in 500 μl of permeabilization solution-I (150 mM sucrose, 80 mM KCl, 35 mM HEPES pH 7.4, 5 mM K_2_HPO_4_, 5 mM MgCl_2_, 0.5 mM CaCl_2_) with 0.05% triton X-100. It was incubated at room temperature for 5 minutes. The cells were centrifuged and washed with permeabilization solution-I without triton X-100 and then resuspended in permeabilization solution-II (150 mM sucrose, 50 mM Tris-Cl pH 7.5, 50 mM NaCl, 2 mM CaCl_2_) containing micrococcal nuclease and incubated for 5 minutes at room temperature. To stop the enzymatic reaction, equal volume of 2X TNESK solution (20 mM Tris Cl pH 7.4, 0.2 M NaCl, 2 mM EDTA, 2% SDS, 0.2 mg/ml proteinase K) and lysis dilution buffer (150 mM NaCl, 5 mM EDTA) was added and incubated at 37°C in water bath for 2 hours. Isolation of MNase digested chromatin from highly permeabilized parasite was done using ethanol precipitation. Two volumes of cold ethanol and 3 M sodium acetate, pH 5.2 (0.3 M final concentration) were added and mixed by inverting and incubated at -20°C for overnight. It was centrifuged at 20,000g for 20 minutes at 4°C. The pellet was washed with 70% ethanol and air dried. The chromatin was dissolved in TE buffer (pH 8.0).

### Histone acetyltransferase activity assay

HAT assay was performed to quantify hyperacetylation by HAT2 over-expressing *L*. *donovani* promastigote cells. Total cell lysate for the assay was prepared by five freeze-thaw cycles in liquid nitrogen vapour phase and then 3 pulses of sonication at 20% efficiency for 20 seconds at an interval of 20 seconds. Total protein in each lysate was quantified using BCA protein assay kit (Pierce). HAT assay was performed using Histone acetyltransferase activity assay kit (Abcam) as per manufacturer’s protocol. OD was measured at 440 nm at an interval of 30 minutes and HAT activity was expressed as relative OD_440nm_/μg/minutes of total protein.

### Western blotting

Western blot was performed using HAT2 over-expressing *L*. *donovani* cells. Cells transfected with pLPNeo2 plasmid without any insert was used as control. 2 x 10^7^ cells were pelleted and resuspended in 100 μl 2X SDS loading dye. 18% SDS PAGE was run and the protein bands were transferred to PVDF membrane. 1:2000 dilution of Anti-acetyl-Histone H4 (Millipore, Cat. 06–866) was used as primary antibody [38]. Anti-rabbit IgG whole molecule, peroxidase antibody (Sigma, Cat. A0545) was used as secondary antibody. The blot was developed using 3, 3’, 5, 5’- Tetramethyl benzidine (Sigma) as substrate. For nuclear loading control, blot was reprobed with anti histone H3 antibody (Abcam, Cat. ab1791).

### Histone H3 total acetylation assay

Histone H3 acetylation was determined using Histone H3 total acetylation detection fast kit (Abcam) by fluorometric method as per manufacturer’s protocol. Isolated histone proteins were used as sample for the assay. Percentage acetylation of HAT2 over-expressing *L*. *donovani* was calculated and compared with acetylation of WT and pLPneo2 vector only transfected *L*. *donovani*.

### Histone H4K4 acetylation assay

Acetylation at lysine-4 of histone H4 was determined using Histone (acetyl K5) quantification kit (Abcam) by colorimetric method. Histone H4K4 acetylation percentage of HAT2 over-expressing *L*. *donovani* was calculated and compared with acetylation of WT and pLPneo2 vector only transfected *L*. *donovani*.

### Statistical methods

The assay results are expressed as the mean ± S.D. The statistical significance of differences in acetylation levels were evaluated by one-way analysis of variance followed by Dunnett’s multiple comparison test. The differences were considered significant if the probability values were <0.05. In MNase digestion assay, two-way analysis of variance followed by Bonferroni post test was applied to check the statistical significance of differences in mononucleosomes/dinucleosomes for WT and HAT2 over-expressing *L*. *donovani* cells.

## Results

### Histone H4 of *L*. *donovani* has conserved N-terminal lysine residues

**Fig 1:**
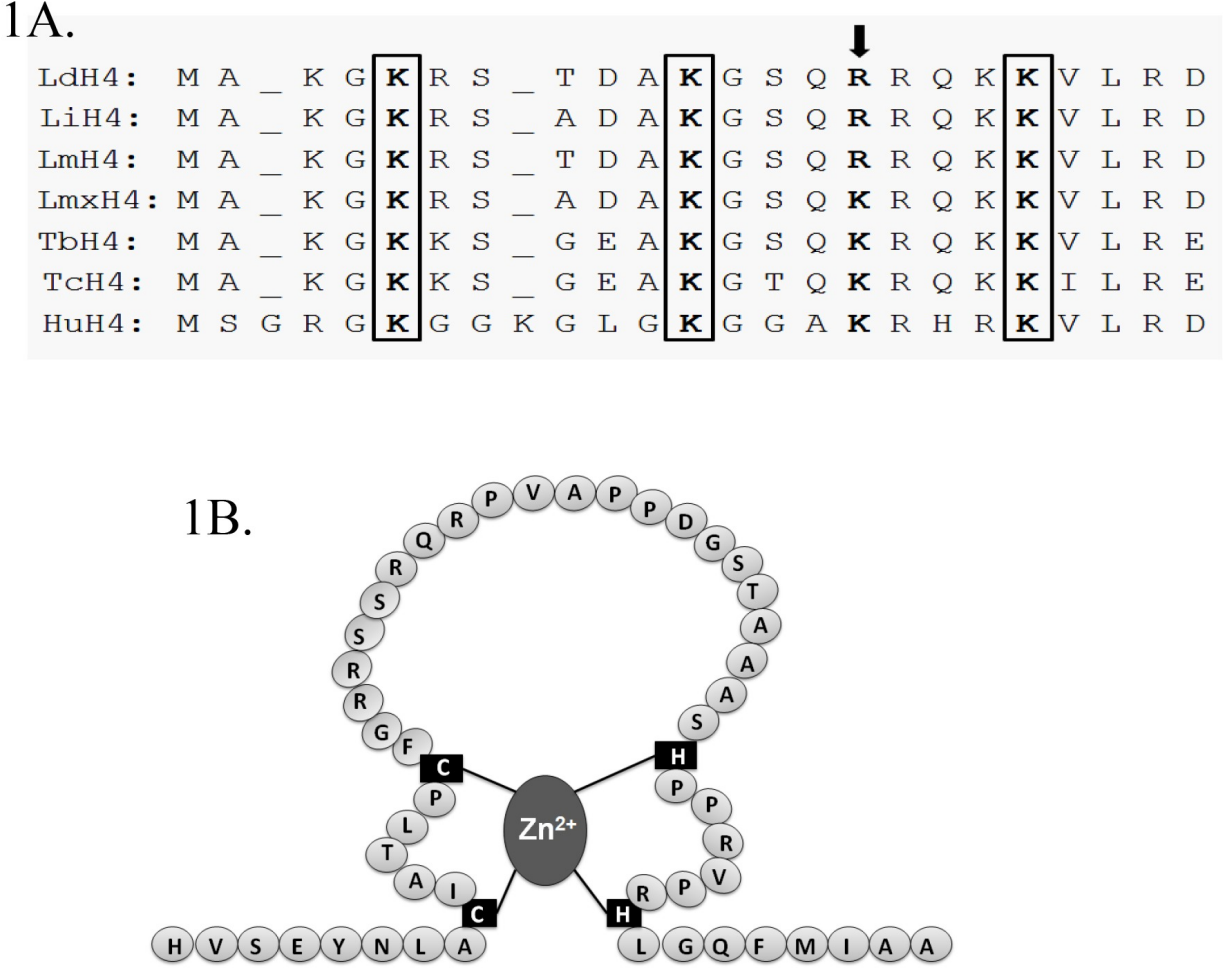
Sequence analysis of Histone H4 and HAT2 of *L*. *donovani*. (1A) Comparison of N-terminus region of histone H4 of *L*. *donovani* (LdH4) with that of *L*. *infantum* (LiH4), *L*. *major* (LmH4), *L*. *mexicana* (LmxH4), *Trypanosoma brucei* (TbH4), *T*. *cruzi* (TcH4) and human (HuH4). Boxes represent conserved lysine residues. The arrow represents K16 of human histone H4 which is replaced by arginine (R14) in *L*. *donovani*. (1B) Zinc finger motif present in the sequence of HAT2 formed by two cysteine and two histidine residues (C_2_H_2_ type).

Histone H4 is among four histones which constitutes eukaryotic nucleosomal core. *Leishmania donovani* histone H4 is a basic protein of ~11.4 kDa. Apart from DNA binding, it has sites for interaction with proteins such as H2A- H2B enabling it to form nucleosomes. The N-terminal tail of histone H4 has been reported to protrude out of nucleosomal core plane which interacts with the linker DNA and acidic patch of H2A-H2B of adjacent nucleosomes to make a condensed chromatin structure [39]. Analysing the N-terminal histone H4 protein from different species of Trypanosomatids and comparing it with human histone H4, it was observed that the lysine residues which are prone to acetylation are conserved. Lysine residues on histone H4 i.e. H4K4, H4K10 and H4K18 in Trypanosomatids correspond to H4K5, K4K12 and H4K20 of human histone H4 respectively (Fig 1A). An important lysine residue in human histone H4 i.e. H4K16 which is mostly acetylated has been replaced by arginine residue (R14) in *L*. *donovani*. These conserved lysine residues along with arginine (H4R14) may have important role in N-terminal tail neutralization of histone H4.

### *Leishmania donovani* HAT2 is MYST family protein with C_2_H_2_ type Zinc finger motif

*L*. *donovani* histone acetyltransferase-2 is a ~67 kDa protein. In the protein sequence, a domain between 181 to 351 amino acid residues shows similarity with human MOZ (monocytic leukaemia zinc finger protein) and SAS (something about silencing) super family proteins. Presence of SAS2 type domain suggests that HAT2 of *Leishmania donovani* is a member of MYST (named after its four founding members i.e. Human MOZ, Yeast Ybf2, Yeast SAS2 and Mammalian TIP60) family protein. In earlier studies, HAT3 and HAT4 of *L*. *donovani* have also been described as members of MYST family. HAT2 has a defined Zinc finger motif which is a characteristic property of MYST family protein. This motif is formed by two cysteine and two histidine residues (C_2_H_2_ type) present in the SAS2 domain region (Fig 1B). Both the cysteines are part of small β-sheets while two histidines in the motif are present in loop and β-sheet structures respectively. Zinc finger motif of HAT2 is apparently associated with DNA binding function similar to that of other members of MYST family.

### Over-expression of HAT2 in *Leishmania donovani*

**Fig 2:**
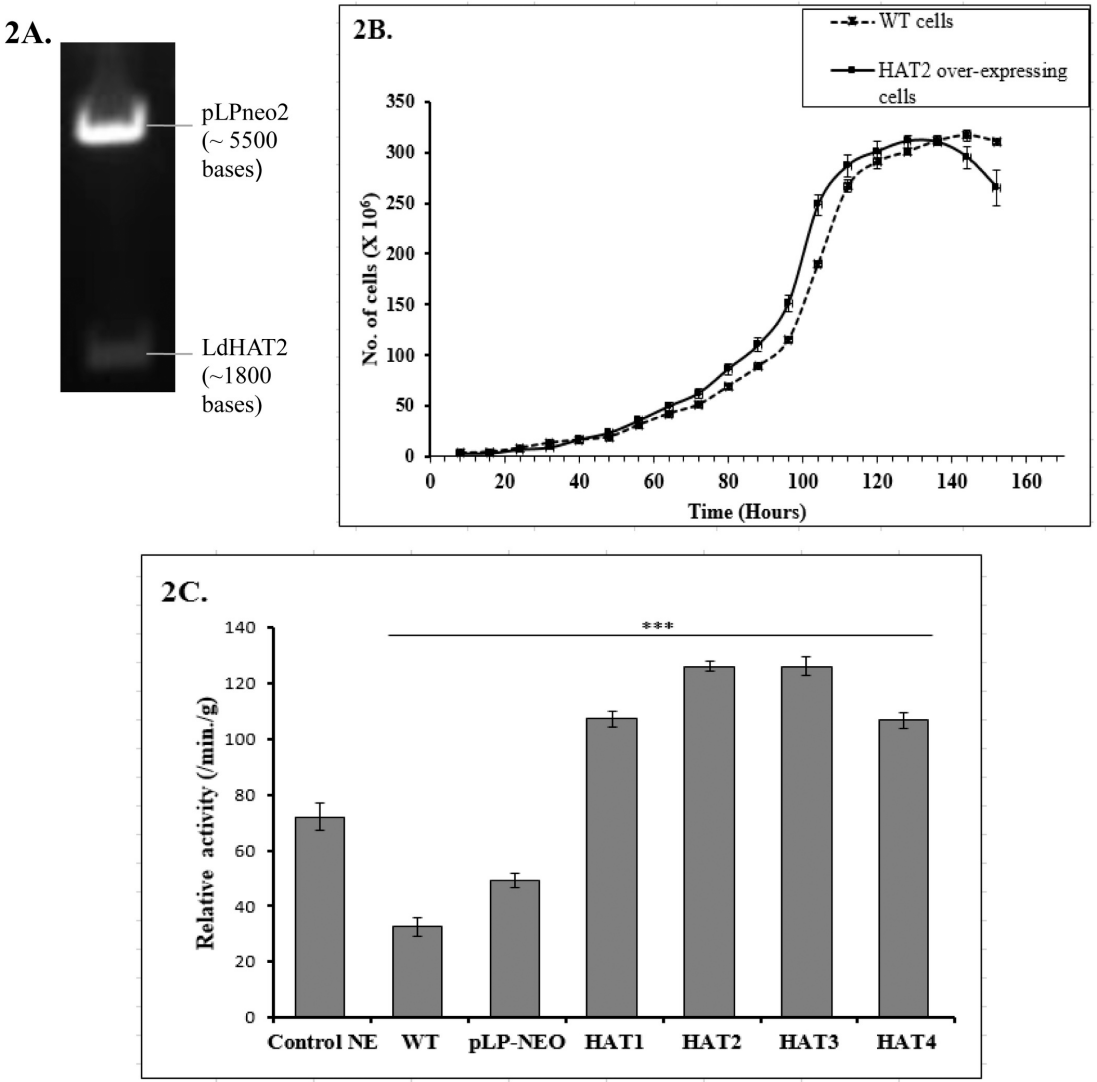
HAT2 over-expression and activity analysis. (2A) RE double digestion of HAT2 cloned in pLPneo2. Clone was digested with HindIII and XhoI. Two fragments (~1800 base pairs for HAT2 and ~5500 base pairs for vector pLPneo2) were resolved in 1% agarose gel. (2B) WT and HAT2 over-expressing *L*. *donovani* cells were counted every eight hours for seven days and plotted. P <0.0001 was observed in two-way analysis of variance showing statistical significance. (2C) Relative histone acetylation activity assay for *L*. *donovani* cells over-expressing HAT1, HAT2, HAT3 and HAT4. The activities were compared to WT cells and vector (pLPneo2) alone transfected cells. Nuclear extract supplied with kit was taken as experimental control. Total histone acetylation was measured at 2 hours 30 minutes and 3 hours after start of reaction and relative activities were calculated per minute and per gram of total cell extract. The data shown represent average of three independent experiments. The p values < 0.0001 compared with control values is shown as ***.

Four different HATs have been reported in trypanosomatids. To study the functional and physiological aspects, HAT2 was over-expressing in *L*. *donovani*. Parallely, other three HATs (HAT1, HAT3 and HAT4) were also over-expressing to perform a comparative study. Here, we have described cloning of only HAT2. The gene coding for HAT2 was amplified by PCR and cloned in pLPneo2 vector in *E*. *coli* DH5α as detailed in materials and methods section. Clones were checked for insert by restriction analysis (Fig 2A). Two bands corresponding to vector (~5.5 kb) and insert (HAT2, ~1800 bases) were observed on 1% agarose gel. Plasmid DNA recovered from confirmed clones was transfected in exponentially growing *L*. *donovani* by electroporation and transfectants were selected in 100μg/ml G418. After three passages, cells were used for further experiments.

### *Leishmania donovani* over-expressing HAT2 exhibits a faster growth

The effect of HAT2 over-expression was studied on growth and culture characteristics of *Leishmania donovani*. The HAT2 over-expressing promastigotes were similar to the WT cells in shape, size and other morphological features such as presence of flagellum. Growth curves of both WT and HAT2 over-expressing *L*. *donovani* were compared by counting the number of live cells every 8 hours. Starting with equal inoculums (2 x 10^5^ cells/ml), both the cultures presented a marked growth difference (5.1 x 10^6^ cells/ml for WT and 6.2 x 10^6^ cells/ml for HAT2 over-expressing cells) after 72 hours. HAT2 over-expressing cells reached log phase sooner than its WT counterpart (Fig 2B).

### All four histone acetyltransferases are functionally active in *L*. *donovani*

HATs are associated with acetylation of different sites of histones, their variants, as well as some non-histone proteins like transcription factors [40]. Four putative histone acetyltransferase genes were found in the genome of *L*. *donovani*. The first and foremost step was to assess whether these genes are coding for functionally active HATs. Acetylation levels of proteins in cell lysates of HAT over-expressing Leishmania were compared. WT and vector alone (pLPneo2) transfected *L*. *donovani* lysates were used as control. The higher levels of acetylation in HAT over-expressing cells indicate that the cloned genes are functionally active in *L*. *donovani*. As compared to WT cells, the over-expressing cells have 3–4 folds higher acetylation (Fig 2C). This indirectly confirmed the over-expression of HATs in *L*. *donovani*.

### HAT2 is involved in histone H4K4 acetylation in Leishmania

**Fig 3:**
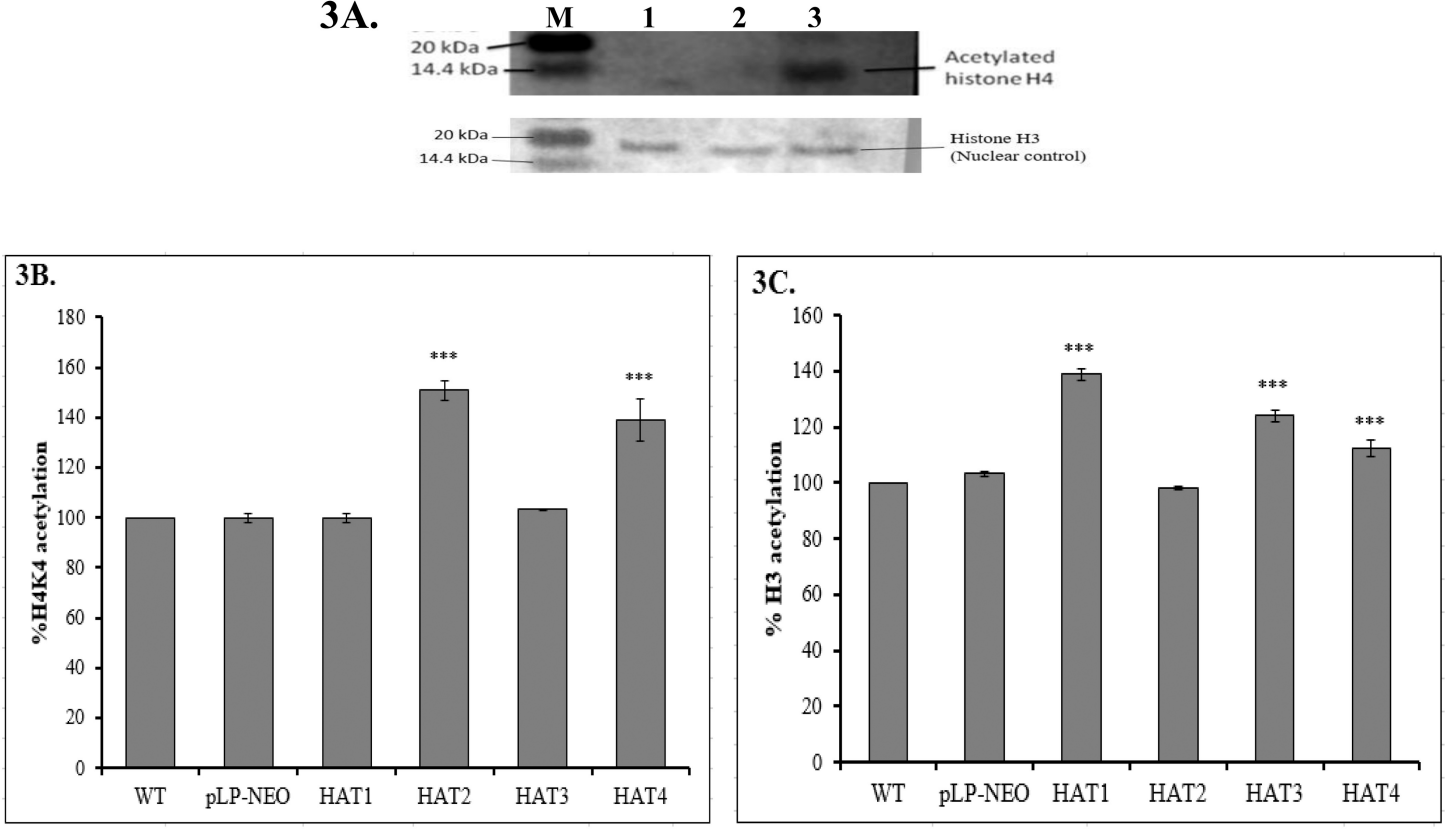
Acetylation of Histones H3 and H4 in over-expressing *Leishmania donovani*. (3A) Western blot analysis of HAT2 over-expressing *L*. *donovani* (Lane 3) using Anti-acetyl-Histone H4 (Millipore, Cat. 06–866) antibody. WT (Lane 1) and vector (pLPneo2) transfected *L*. *donovani* (Lane 2) were used as controls. M indicates molecular weight marker. For nuclear loading control, blot was reprobed with anti H3 antibody. (3B) H4K4 acetylation assay using histones isolated from HATs over-expressing cells by colorimetric method. Histones of WT cells and pLPneo2 transfected cells were used as control. Relative levels of acetylation of H4K4 are shown in percentage assuming that of WT cells as 100%. The p values < 0.0001 compared with WT cells are shown as ***. (3C) Total histone H3 acetylation assay by fluorimetric method for HATs over-expressing cells. Histones isolated from WT cells and vector (pLPneo2) only transfected cell were taken as controls. H3 acetylation was presented in percentage considering 100% for WT cells. *** represents p values < 0.0001 compared to WT cells.

After observing overall higher levels of acetylation of various HAT over-expressing *L*. *donovani* lysates, it was tempting to check the acetylation of core histones H3/H4 and its effects on chromatin. In earlier studies *L*. *donovani* HAT1, HAT3 and HAT4 have been shown to mediate acetylation of histone H4K10, proliferating cell nuclear antigen (PCNA) and histone H4K14 respectively [32, 33, 41]. Therefore, our aim was to explore the acetylation site for HAT2 within core histones H3/H4 of *L*. *donovani* and study its functional aspects. The Western blot analysis of HAT2 over-expressing cells with Anti-acetyl-Histone H4 antibodies suggested an increased acetylation of histone H4 (Fig 3A). Again, it was interesting to investigate the histone H4 sites at which specifically HAT2 was acetylating. The terminal lysine 4 of histone H4 was most important site for acetylation as the terminal portion is involved in interaction with the linker DNA as well as with other nucleosomes. Also, in *T*. *brucei* it is the primary acetylation site of histone H4 [27]. To understand the H4K4 acetylation, histones were isolated from the HAT over-expressing *L*. *donovani* and used in colorimetric assay. In *Trypanosoma brucei*, it has been shown that H4K4 acetylation is mediated by HAT3 [42]. In contrast, H4K4 acetylation remained unaffected by HAT3 over-expression in *L*. *donovani*. HAT2 over-expressing cells showed a significantly higher H4K4 acetylation than WT and only vector (pLPneo2) transfected cells (Fig 3B).

### HAT2 doesn’t affect histone H3 acetylation in Leishmania

Histone H3 acetylation is considered important for linker DNA length and organisation of chromatin [43]. Therefore, it was necessary to check histone H3 acetylation pattern for HAT2 over-expressing cells. A fluorimetric assay was performed using isolated histones from HATs over-expressing *L*. *donovani* cells. It was observed that out of four HATs present in Leishmania, only HAT2 was not involved in histone H3 acetylation (Fig 3C). Other three histones i.e. HAT1, HAT3 and HAT4 were more or less acetylating Leishmania histone H3.

### MNase digestion of HAT2 over-expressing *L*. *donovani* chromatin generates more mononucleosomes and dinucleosomes

The histone acetylation pattern of HAT2 over-expressing *L*. *donovani* cells suggested its specific involvement in histone H4K5 acetylation. This neutralization of charge on the terminal part of histone H4 tail may have a number of functional attributes. The most important effect was expected on inter-nucleosomal and/or linker DNA-core interactions which are responsible for condensation of chromatin structure. Therefore, it became necessary to analyse the condensation of chromatin for which indirect method of MNase digestion was used.

**Fig 4:**
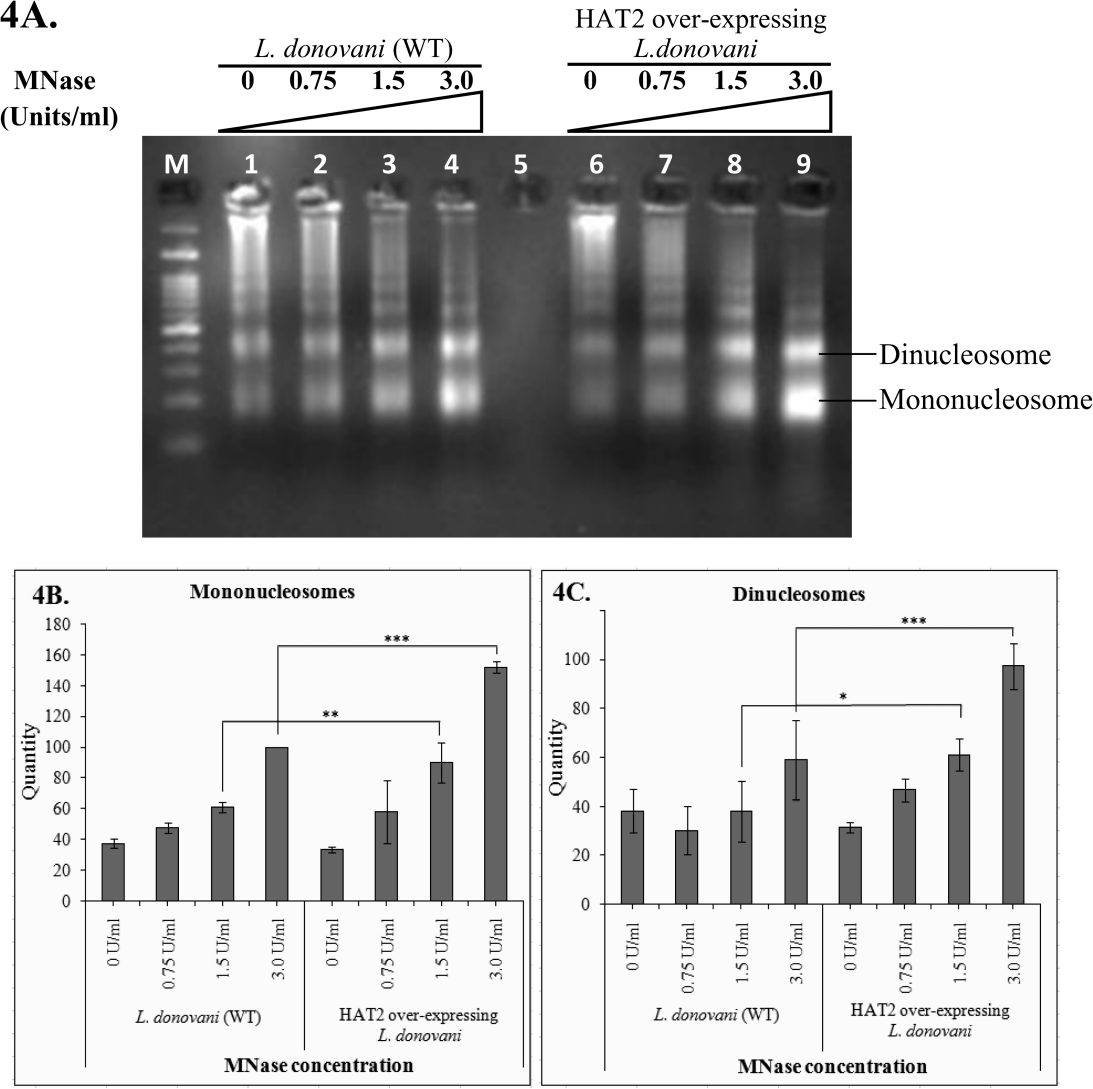
MNase digestion of WT and HAT2 over-expressing *Leishmania donovani* chromatin. (4A) *L*. *donovani* cells (WT and HAT2 over-expressing) were permeabilized with Triton X-100 and treated with varying units of MNase (0.75, 1.5 and 3.0 U/ml) and chromatin was isolated and run on 3% agarose gel. Untreated permeabilized WT and HAT2 overexpressed cells were processed identically (Lane 1 and Lane 6 respectively). In Lane M, molecular weight marker (100 bp ladder) was run. Positions of mononucleosomes and dinucleosomes were indicated for comparing their abundances. (4B) Intensities of bands corresponding to mononucleosomes were quantified using Gene Tools software (Syngene). Statistical significance was checked by two-way analysis of variance followed by Bonferroni post test. ** and *** represents p values < 0.01 and < 0.001, respectively compared to WT cells. (4C) Comparison of dinucleosomal band intensities in WT and HAT2 over-expressing *L*. *donovani* cells at varying MNase concentrations (0.75, 1.5 and 3.0 U/ml). Two-way analysis of variance followed by Bonferroni post test was applied to check the statistical significance of differences of band intensities between WT and HAT2 over-expressing cells. P values <0.05 and <0.001 is represented by * and ***, respectively.

Permeabilized WT and HAT2 over-expressing *L*. *donovani* cells were treated with increasing concentrations of MNase (i.e. 0.75, 1.5 and 3.0 units/ml) for five minutes. The cells that were permeabilized but not treated with MNase were processed as control. Treated and control cells were subjected to chromatin extraction. Abundance of mononucleosomes and dinucleosomes were compared by analysing the extracted DNA on 3% agarose gel. HAT2 over-expressing cells showed significantly higher mono and dinucleosomes than WT *L*. *donovani* cells for respective MNase concentrations (Fig 4A). At 3.0 U/ml MNase concentration, the mononucleosomes were 53.33% more (Fig 4B) whereas, dinucleosomes were 65.26% more (Fig 4C) in HAT2 over-expressing cells as compared to WT *L*. *donovani* cells. Higher intensity of mononucleosome and dinucleosome advocates that the chromatin is more accessible to MNase and thus correlates with more decondensed chromatin.

This suggests an important role of *Leishmania donovani* Histone acetyltransferase-2 (HAT2) in regulation of chromatin condensation.

## Discussion

Alteration in chromatin architecture is the main focus of epigenetic studies. Variation in inter-nucleosomal linker DNA, incorporation of core histone variants and post translational modifications of histones at N-terminal tail regions are the key factors affecting chromatin structure [22, 44, 45]. Additionally, chromatin architectural proteins (CAPs) such as MeCP2, MENT, polycomb and HP1α have also been shown to modulate chromatin conformation [46]. Presence of genes encoding epigenetic determinants along with variants of histones and wide range of histone modifying enzymes in *Leishmania donovani* genome suggests an important role of chromatin alteration in its survival and pathogenicity. The preliminary studies involving histone modifiers such as HAT1, HAT3 and HAT4 have initiated exploring epigenetic involvement in various processes [33, 41]. Protein sequence analysis suggests that *L*. *donovani* HAT2 belongs to MYST family of histone acetyltransferase. MYST family proteins are conserved in eukaryotic organisms and are marked for nuclear acetylations [47]. The SAS2 type domain found in HAT2 may be vital for Leishmania as it has proven transcriptional silencing activity in yeast [48]. It will be interesting to see the effect of HAT2 deletion on survival of *L*. *donovani* as in *T*. *brucei*, the attempts to produce HAT2 null mutant turned futile suggesting that HAT2 is essential for its growth [49].

The analysis of Leishmania genome revealed presence of four histone acetyltransferase genes. For the first time we are reporting that all four HATs are actively acetylating histones in *L*. *donovani*. Although, histone acetylation activity was quantitatively similar for both HAT2 and HAT3, the physiological relevance may be completely distinct depending on the acetylation of specific residues at different histones. But the possibility of crosstalk between different HATs may not be ruled out as the target amino acids for acetylation may be shared by HATs. Present study is focused on HAT2 mediated acetylation, its consequent chromatin condensation and possible transcriptional silencing. The HAT2 over-expressing *L*. *donovani* indicated higher acetylation on histone H4. But, the target amino acids for acetylation on N-terminal H4 tail by HAT2 needed to be determined. The protruding tail region of H4 histone is thought to be smaller than that of H3. Hence, the PTMs at residues on terminal part of histone H4 tail may have larger role to play in nucleosomal interactions. HAT2 over-expressing cells showed a significantly higher H4K4 acetylation in *L*. *donovani* which was in contrast to *T*. *brucei* where the H4K4 acetylation is carried out by HAT3 [42]. Further, H4K4 acetylation in *L*. *donovani* remained unaffected on HAT3 over-expression.

H4K4 in *L*. *donovani* is analogous to mammalian H4K5 which has been shown to be associated with differential gene expression in mice. H4K5 acetylation near promoter as well as coding regions has been linked with upregulation of gene expression [50]. It may allow higher access of RNA polymerase II and other transcription factors to their respective binding sites. In Leishmania, we have applied MNase digestion to assess the effect of H4K4 acetylation on chromatin condensation. Results showed that HAT2 over-expressing Leishmania cells have higher proportion of mononucleosomes and dinucleosomes which is an indication of chromatin decondensation. Though our result shows that HAT4 carries out H4K4 acetylation, its effect on chromatin condensation is not that profound. This is probably due to cytoplasmic localization of HAT4 where it might acetylate free histones [33, 51]. Effect of chromatin decondensation may result in changes in transcription of unidirectional gene arrays of *L*. *donovani* which needs to be investigated. We have shown that growth of HAT2 over-expressing protozoan parasite was faster than its wild-type counterpart. This may be an outcome of decondensed chromatin and consequent changes in gene transcription profile.

Our work has explored the epigenetic involvement of HAT2 in chromatin compactness of *Leishmania donovani*. Being a digenetic parasite, Leishmania encounters drastic changes in its microenvironment during its life cycle in two hosts, sand fly and human. It may involve intricate regulation of stage specific gene expression that remains unhighlighted at large. Therefore, it is important to understand the role of alterations at chromatin level on global regulation of transcription. Same should also be examined in the context of divergent strand switch region which is thought to have regulatory regions for gene arrays. The epigenetic regulators affecting basic architecture of chromatin may be exploited for designing newer therapeutic strategies against leishmaniasis as the existing drugs are suffering from toxicity and drug resistance issues.

## Conclusion

Leishmania is a digenetic protozoan parasite which completes its life cycle alternating between human and sand fly. Host transition is accompanied by up/down regulation of various genes. The mechanism by which this drastic change in expression profile is regulated remains largely unknown and we propose the involvement of epigenetic determinants in this process. Studies in other organisms have established that transcription is dependent on the accessibility of sites on DNA to transcription machinery, transcription factors and other regulators. We have demonstrated the involvement of HAT2, a MYST family protein, in modulation of chromatin accessibility in *Leishmania donovani*. HAT2 mediates acetylation of histone H4 on lysine-4 neutralizing its positive charge on amino terminus protruding tail. This weakens the interaction of histone H4 with neighbouring nucleosomes and linker DNA, resulting into loosely packed nucleosomes. This was supported by our data from MNase digestion of chromatin that shows higher accessibility in HAT2 over-expressing *L*. *donovani*. This suggests the potential role of HAT2 in global regulation of transcription in *L*. *donovani*. HAT2 over-expressing *L*. *donovani* grows faster than its WT counterpart which may be an outcome of transcriptional changes due to decondensed chromatin. Better understanding of these epigenetic determinants of parasite would help in designing novel therapeutic strategies.

## Supporting information

S1 Fig Western blot analysis for detection of acetylated histone H4 in HAT2 over-expressing *L*. *donovani*. Anti-acetyl histone H4 antibody (Millipore, Cat. 06–866) was used to probe acetylated histone H4. WT (Lane 1) and vector (pLPneo2) only transfected *L*. *donovani* (Lane 2) were used as controls. HAT2 over-expressing *L*. *donovani* cell lysate was loaded in lane 3. M indicates molecular weight marker. Anti-histone H3 antibody (Abcam, Cat. ab1791) was used to reprobe H3, the nuclear loading control.
(TIF)

S2 Fig Schematic presentation of primers used for confirmation of presence of the recombinant plasmid in transfected cells. For_v = Vector specific forward primer, For_g = Gene specific forward primer and Rev_g = Gene specific reverse primers. Primers For_v and Rev_g amplify DNA of 225 bp (only when recombinent plasmid is present in template); whereas For_g and Rev_g generate 175 bp amplicon (from genomic DNA as well as gene present in recombinant plasmid).
(TIF)

S3 Fig Confirmation of HAT2 clone transfectants by PCR. M indicates 100 bp DNA ladder. Lane 1 and 2 contain PCR products using *L*. *donovani* genomic DNA where as Lane 3 and 4 contain that using vector pLPneo2. Lanes 5 and 6 represent PCR amplification using plasmid recovered from HAT2 over-expressing cells.
PCR product of size ~175 bp was amplified using *L*. *donovani* genomic DNA. Same product was observed in amplification with plasmid recovered from HAT2 over-expressing cells and it indicates the presence of HAT2 gene. PCR product of ~225 bp using plasmid recovered from HAT2 over-expressing cells is present and it would appear only when HAT2 is cloned into vector pLPneo2.
(TIF)

S4 Fig Histone isolated from *L*. *donovani* promastigotes. Lane 1–3 represents histones isolated from approximately ~2 x 10^6^ un-transfected, vector (pLPneo2) alone transfected and HAT2 over-expressing promastigotes, respectively.
(TIF)

S1 Appendix Growth curve data. (DOC)

S2 Appendix HAT assay data. (DOC)

S3 Appendix H4K4 acetylation assay data. (DOC)

S4 Appendix Total H3 acetylation data. (DOC)

S5 Appendix Densitometric quantification of Mononucleosomes and Dinucleosomes from MNase digested chromatin. (DOC)
