# Supplementary figures and images for: HAT2 mediates histone H4K4 acetylation and affects micrococcal nuclease sensitivity of chromatin in Leishmania donovani
Source: PLoS One. 2017 May 9;12(5):e0177372. doi: 10.1371/journal.pone.0177372 (PMC5423686; doi:10.1371/journal.pone.0177372)

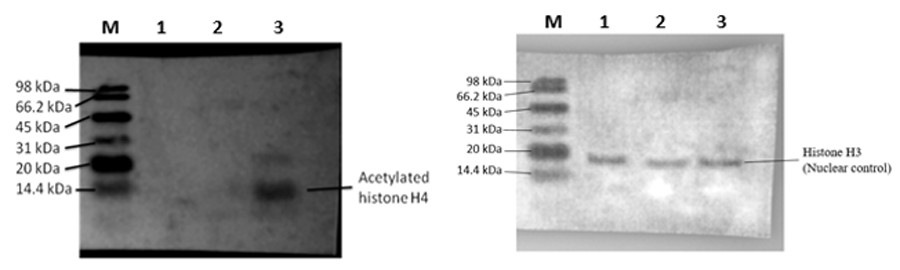

Supplement: S1 Fig — Anti-acetyl histone H4 antibody (Millipore, Cat. 06–866) was used to probe acetylated histone H4. WT (Lane 1) and vector (pLPneo2) only transfected L. donovani (Lane 2) were used as controls. HAT2 over-expressing L. donovani cell lysate was loaded in lane 3. M indicates molecular weight marker. Anti-histone H3 antibody (Abcam, Cat. ab1791) was used to reprobe H3, the nuclear loading control. (TIF) [file pone.0177372.s001.tif]

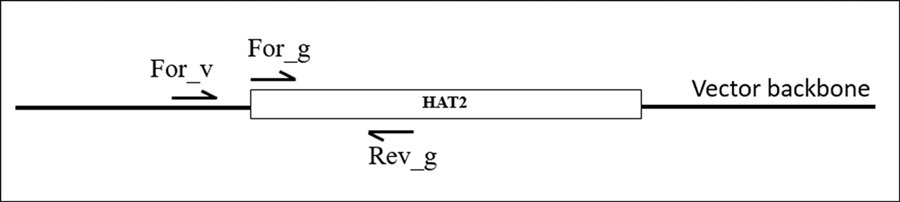

Supplement: S2 Fig — For_v = Vector specific forward primer, For_g = Gene specific forward primer and Rev_g = Gene specific reverse primers. Primers For_v and Rev_g amplify DNA of 225 bp (only when recombinent plasmid is present in template); whereas For_g and Rev_g generate 175 bp amplicon (from genomic DNA as well as gene present in recombinant plasmid). (TIF) [file pone.0177372.s002.tif]

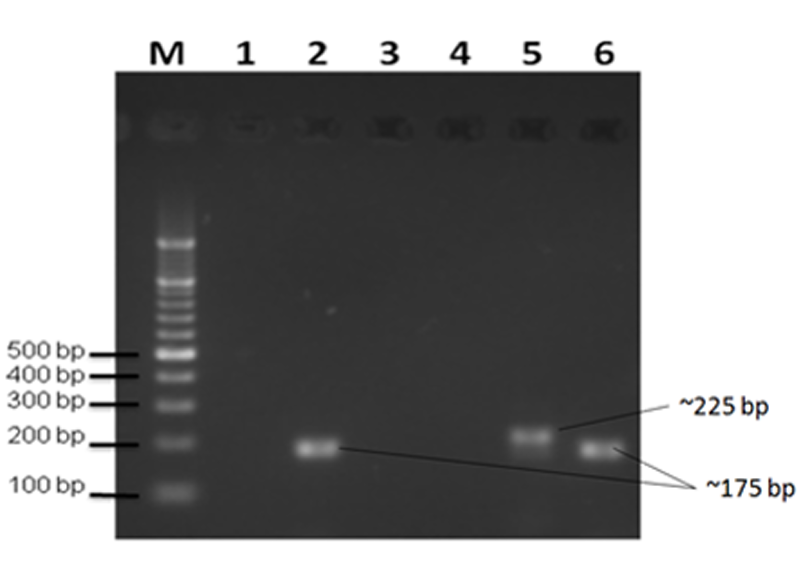

Supplement: S3 Fig — M indicates 100 bp DNA ladder. Lane 1 and 2 contain PCR products using L. donovani genomic DNA where as Lane 3 and 4 contain that using vector pLPneo2. Lanes 5 and 6 represent PCR amplification using plasmid recovered from HAT2 over-expressing cells. PCR product of size ~175 bp was amplified using L. donovani genomic DNA. Same product was observed in amplification with plasmid recovered from HAT2 over-expressing cells and it indicates the presence of HAT2 gene. PCR product of ~225 bp using plasmid recovered from HAT2 over-expressing cells is present and it would appear only when HAT2 is cloned into vector pLPneo2. (TIF) [file pone.0177372.s003.tif]

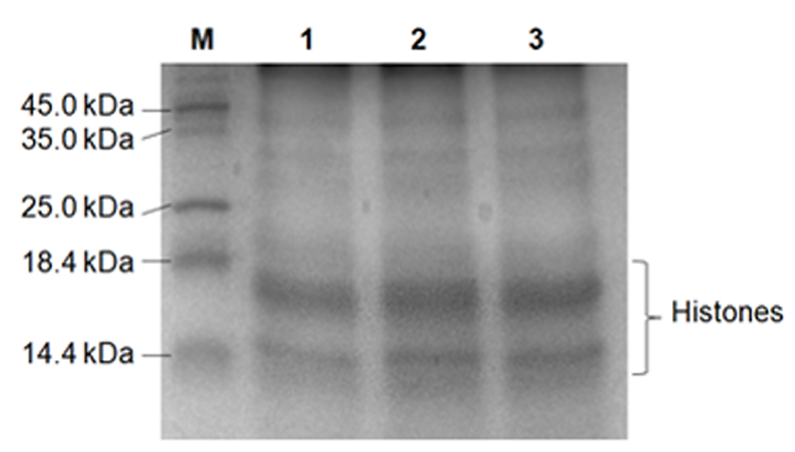

Supplement: S4 Fig — Lane 1–3 represents histones isolated from approximately ~2 x 10^6^ un-transfected, vector (pLPneo2) alone transfected and HAT2 over-expressing promastigotes, respectively. (TIF) [file pone.0177372.s004.tif]
