# Supplementary material for: HAT2 mediates histone H4K4 acetylation and affects micrococcal nuclease sensitivity of chromatin in Leishmania donovani
Source: PLoS One. 2017 May 9;12(5):e0177372. doi: 10.1371/journal.pone.0177372 (PMC5423686; doi:10.1371/journal.pone.0177372)
Supplement: S1 Appendix — (DOC) [file pone.0177372.s005.doc]

**S1 Appendix: Growth Curve Data**

| **Time (Hours)** | **WT *Leishmania donovani***  **(Cells x 10^6^)** | | | | **HAT2 over-expressing *Leishmania donovani***  **(Cells x 10^6^)** | | | |
| --- | --- | --- | --- | --- | --- | --- | --- | --- |
|  | **I** | **II** | **II** | **Average** | **I** | **II** | **III** | **Average** |
| 8 | 2.0 | 3.0 | 4.0 | 3.0 | 2.0 | 2.0 | 2.0 | 2.0 |
| 16 | 4.0 | 4.0 | 4.0 | 4.0 | 2.0 | 3.0 | 2.0 | 2.3 |
| 24 | 8.0 | 7.0 | 9.0 | 8.0 | 5.0 | 7.0 | 6.0 | 6.0 |
| 32 | 13.0 | 12.0 | 14.0 | 13.0 | 7.0 | 9.0 | 8.0 | 8.0 |
| 40 | 16.0 | 16.0 | 16.0 | 16.0 | 14.0 | 17.0 | 17.0 | 16.0 |
| 48 | 18.0 | 19.0 | 20.0 | 19.0 | 20.0 | 24.0 | 24.0 | 22.7 |
| 56 | 31.0 | 30.0 | 32.0 | 31.0 | 32.0 | 37.0 | 36.0 | 35.0 |
| 64 | 42.0 | 41.0 | 43.0 | 42.0 | 45.0 | 52.0 | 50.0 | 49.0 |
| 72 | 50.0 | 52.0 | 51.0 | 51.0 | 57.0 | 65.0 | 64.0 | 62.0 |
| 80 | 67.0 | 71.0 | 70.0 | 69.3 | 80.0 | 90.0 | 89.0 | 86.3 |
| 88 | 88.0 | 90.0 | 89.0 | 89.0 | 102.0 | 116.0 | 112.0 | 110.0 |
| 96 | 113.0 | 117.0 | 115.0 | 115.0 | 141.0 | 156.0 | 156.0 | 151.0 |
| 104 | 188.0 | 191.0 | 192.0 | 190.3 | 237.0 | 252.0 | 256.0 | 248.3 |
| 112 | 261.0 | 272.0 | 268.0 | 267.0 | 275.0 | 295.0 | 291.0 | 287.0 |
| 120 | 283.0 | 298.0 | 292.0 | 291.0 | 290.0 | 307.0 | 306.0 | 301.0 |
| 128 | 297.0 | 303.0 | 303.0 | 301.0 | 304.0 | 313.0 | 316.0 | 311.0 |
| 136 | 306.0 | 315.0 | 314.0 | 311.7 | 311.0 | 307.0 | 312.0 | 310.0 |
| 144 | 311.0 | 319.0 | 321.0 | 317.0 | 308.0 | 287.0 | 290.0 | 295.0 |
| 152 | 311.0 | 309.0 | 313.0 | 311.0 | 285.0 | 257.0 | 253.0 | 265.0 |
