# Supplementary material for: HAT2 mediates histone H4K4 acetylation and affects micrococcal nuclease sensitivity of chromatin in Leishmania donovani
Source: PLoS One. 2017 May 9;12(5):e0177372. doi: 10.1371/journal.pone.0177372 (PMC5423686; doi:10.1371/journal.pone.0177372)
Supplement: S2 Appendix — (DOC) [file pone.0177372.s006.doc]

**S2** Appendix: HAT Assay Data

| **EXPERIMENT 1** | |  |  |  |  | | |  | | |  | |  | | |
| --- | --- | --- | --- | --- | --- | --- | --- | --- | --- | --- | --- | --- | --- | --- | --- |
|  | | | **Total protein in lysate (µg/µl)** | **Average OD at 440 nm** | | | | **OD_440_**  **(3hr-2.5hr)** | | **Subtracting background** | | **Relative activity (/min./µg)** | | |  |
|  |  |  |  | **3 hour** | | **2.5 hour** | |  |  |  |  |  |  |  |  |
| 1 | (-) ve control | 40 µl water | 0 | 0.437 | | 0.353 | | 0.084 | |  | |  | | |  |
| 2 | (+) ve control | 10 µl NE + 30 µl water | 50 | 1.171 | | 0.985 | | 0.186 | | 0.102 | | 0.000068 | | |  |
| 3 | Ag83 | 10 µl Sample + 30 µl water | 24.58 | 0.598 | | 0.492 | | 0.106 | | 0.022 | | 2.98346E-05 | | |  |
| 4 | pLPneo2 | 10 µl Sample + 30 µl water | 24.95 | 0.542 | | 0.423 | | 0.119 | | 0.035 | | 4.70274E-05 | | |  |
| 5 | HAT1 | 10 µl Sample + 30 µl water | 24.24 | 0.506 | | 0.346 | | 0.160 | | 0.076 | | 0.00010396 | | |  |
| 6 | HAT2 | 10 µl Sample + 30 µl water | 24.82 | 0.594 | | 0.417 | | 0.177 | | 0.093 | | 0.000124886 | | |  |
| 7 | HAT3 | 10 µl Sample + 30 µl water | 23.01 | 0.552 | | 0.383 | | 0.169 | | 0.085 | | 0.00012328 | | |  |
| 8 | HAT4 | 10 µl Sample + 30 µl water | 23.42 | 0.562 | | 0.405 | | 0.157 | | 0.073 | | 0.000104184 | | |  |
|  |  |  |  |  | |  | |  | | |  | |  | | |
|  |  |  |  |  | |  | |  | | |  | |  | | |
| **EXPERIMENT 2** | |  |  |  | |  | |  | | |  | |  | | |
|  | | | **Total protein in lysate (µg/µl)** | **Average OD at 440 nm** | | | | **OD_440_ (3hr-2.5hr)** | | | **Subtracting background** | | | **Relative activity (/min./µg)** | |
|  |  |  |  | **3 hour** | | **2.5 hour** | |  |  |  |  |  |  |  |  |
| 1 | (-) ve control | 40 µl water | 0 | 0.532 | | 0.437 | | 0.095 | | |  | | |  | |
| 2 | (+) ve control | 10 µl NE + 30 µl water | 50 | 1.237 | | 1.036 | | 0.201 | | | 0.106 | | | 7.06667E-05 | |
| 3 | Ag83 | 10 µl Sample + 30 µl water | 24.58 | 0.632 | | 0.514 | | 0.118 | | | 0.023 | | | 3.15975E-05 | |
| 4 | pLPneo2 | 10 µl Sample + 30 µl water | 24.95 | 0.592 | | 0.460 | | 0.132 | | | 0.037 | | | 4.90314E-05 | |
| 5 | HAT1 | 10 µl Sample + 30 µl water | 24.24 | 0.536 | | 0.363 | | 0.174 | | | 0.079 | | | 0.000107948 | |
| 6 | HAT2 | 10 µl Sample + 30 µl water | 24.82 | 0.622 | | 0.434 | | 0.189 | | | 0.094 | | | 0.000125598 | |
| 7 | HAT3 | 10 µl Sample + 30 µl water | 23.01 | 0.578 | | 0.397 | | 0.181 | | | 0.086 | | | 0.000125018 | |
| 8 | HAT4 | 10 µl Sample + 30 µl water | 23.42 | 0.585 | | 0.415 | | 0.170 | | | 0.075 | | | 0.000106462 | |
|  |  |  |  |  | |  | |  | | |  | | |  | |
|  |  |  |  |  | |  | |  | | |  | | |  | |
| **EXPERIMENT 3** | |  |  |  | |  | |  | | |  | | |  | |
|  | | | **Total protein in lysate (µg/µl)** | **Average OD at 440 nm** | | | | **OD_440_**  **(3hr-2.5hr)** | | | **Subtracting background** | | | **Relative activity (/min./µg)** | |
|  |  |  |  | **3 hour** | | **2.5 hour** | |  |  |  |  |  |  |  |  |
| 1 | (-) ve control | 40 µl water | 0 | 0.516 | | 0.402 | | 0.114 | | |  | | |  | |
| 2 | (+) ve control | 10 µl NE + 30 µl water | 50 | 1.315 | | 1.085 | | 0.230 | | | 0.116 | | | 7.73333E-05 | |
| 3 | Ag83 | 10 µl Sample + 30 µl water | 24.58 | 0.712 | | 0.571 | | 0.141 | | | 0.027 | | | 3.62083E-05 | |
| 4 | pLPneo2 | 10 µl Sample + 30 µl water | 24.95 | 0.591 | | 0.438 | | 0.153 | | | 0.039 | | | 5.22378E-05 | |
| 5 | HAT1 | 10 µl Sample + 30 µl water | 24.24 | 0.615 | | 0.421 | | 0.194 | | | 0.080 | | | 0.000109873 | |
| 6 | HAT2 | 10 µl Sample + 30 µl water | 24.82 | 0.657 | | 0.448 | | 0.209 | | | 0.095 | | | 0.000128243 | |
| 7 | HAT3 | 10 µl Sample + 30 µl water | 23.01 | 0.625 | | 0.422 | | 0.204 | | | 0.090 | | | 0.000129799 | |
| 8 | HAT4 | 10 µl Sample + 30 µl water | 23.42 | 0.636 | | 0.445 | | 0.191 | | | 0.077 | | | 0.000109593 | |
|  |  |  |  |  | |  | |  | | |  | | |  | |
|  |  |  |  |  | |  | |  | | |  | | |  | |
|  |  |  |  |  | |  | |  | | |  | | |  | |
|  | | | **Total protein in lysate (µg/µl)** | **OD_440_ (3hr-2.5hr)** | | | | | | **Average OD of 3 independent experiments** | | **Relative activity (/min./µg)** | | |  |
|  |  |  |  | **I** | | | **II** | | **III** |  |  |  |  |  |  |
| 1 | (-) ve control | 40 µl water | 0 |  | | |  | |  |  | |  | | |  |
| 2 | (+) ve control | 10 µl NE + 30 µl water | 50 | 0.102 | | | 0.106 | | 0.116 | 0.108 | | 0.000072 | | |  |
| 3 | Ag83 | 10 µl Sample + 30 µl water | 24.58 | 0.022 | | | 0.023 | | 0.027 | 0.024 | | 3.25468E-05 | | |  |
| 4 | pLPneo2 | 10 µl Sample + 30 µl water | 24.95 | 0.035 | | | 0.037 | | 0.039 | 0.037 | | 4.94322E-05 | | |  |
| 5 | HAT1 | 10 µl Sample + 30 µl water | 24.24 | 0.076 | | | 0.079 | | 0.080 | 0.078 | | 0.000107261 | | |  |
| 6 | HAT2 | 10 µl Sample + 30 µl water | 24.82 | 0.093 | | | 0.094 | | 0.095 | 0.094 | | 0.000126242 | | |  |
| 7 | HAT3 | 10 µl Sample + 30 µl water | 23.01 | 0.085 | | | 0.086 | | 0.090 | 0.087 | | 0.000126032 | | |  |
| 8 | HAT4 | 10 µl Sample + 30 µl water | 23.42 | 0.073 | | | 0.075 | | 0.077 | 0.075 | | 0.000106746 | | |  |
|  |  |  |  |  | | |  | |  |  | |  | | |  |
|  |  |  |  |  | | |  | |  |  | |  | | |  |
|  |  |  |  |  | | |  | |  |  | |  | | |  |
|  |  |  |  |  | | |  | |  |  | |  | | |  |
|  |  |  |  |  | | |  | |  |  | |  | | |  |
|  |  | **Relative activity (/min./µg)** | **Relative activity (/min./g)** | | | |  | |  |  | |  | | |  |
|  | Control NE | 0.000072 | 72 | | | |  | |  |  | |  | | |  |
|  | WT | 3.25468E-05 | 32.546786 | | | |  | |  |  | |  | | |  |
|  | pLPneo2 | 4.94322E-05 | 49.43219773 | | | |  | |  |  | |  | | |  |
|  | HAT1 | 0.000107261 | 107.2607261 | | | |  | |  |  | |  | | |  |
|  | HAT2 | 0.000126242 | 126.2422777 | | | |  | |  |  | |  | | |  |
|  | HAT3 | 0.000126032 | 126.0321599 | | | |  | |  |  | |  | | |  |
|  | HAT4 | 0.000106746 | 106.7463706 | | | |  | |  |  | |  | | |  |
