# Supplementary material for: HAT2 mediates histone H4K4 acetylation and affects micrococcal nuclease sensitivity of chromatin in Leishmania donovani
Source: PLoS One. 2017 May 9;12(5):e0177372. doi: 10.1371/journal.pone.0177372 (PMC5423686; doi:10.1371/journal.pone.0177372)
Supplement: S3 Appendix — (DOC) [file pone.0177372.s007.doc]

**S3 Appendix: H4K4 Acetylation Assay Data**

| **EXPERIMENT 1** | | |  |  |  | |  |  |  | |  |  |  |
| --- | --- | --- | --- | --- | --- | --- | --- | --- | --- | --- | --- | --- | --- |
|  |  | **OD at 450 nm** | **Sample OD-Blank OD** |  |  | |  |  |  | |  |  |  |
| Blank |  | 0.302 |  |  |  | |  |  |  | |  |  |  |
| Standard | 100ng/µl | 2.093 | 1.791 |  |  | | **Total histone (µg/µl)** | **OD at 450 nm** | **Sample OD-Blank OD** | | **Slope = 0.009** | **% H4K4ac** | **increase in %H4K4ac** |
|  | 50ng/µl | 0.742 | 0.44 |  |  |  |  |  |  |  | **Amount H4K4ac** |  |  |
|  | 25ng/µl | 0.558 | 0.256 |  | Ag83 | | 1.3365 | 0.504 | 0.202 | | 16.793 | 100.00 |  |
|  | 12.5ng/µl | 0.432 | 0.13 |  | pLPneo2 | | 1.6445 | 0.546 | 0.244 | | 16.486 | 98.17 | -1.831186463 |
|  | 6.25ng/µl | 0.406 | 0.104 |  | HAT1 | | 1.6885 | 0.555 | 0.253 | | 16.649 | 99.14 | -0.862708421 |
| Ag83 |  | 0.504 | 0.202 |  | HAT2 | | 1.628 | 0.663 | 0.361 | | 24.638 | 146.71 | 46.71360717 |
| pLPneo2 |  | 0.546 | 0.244 |  | HAT3 | | 1.353 | 0.512 | 0.21 | | 17.246 | 102.69 | 2.692586332 |
| HAT1 |  | 0.555 | 0.253 |  | HAT4 | | 1.5015 | 0.637 | 0.335 | | 24.790 | 147.62 | 56.43020346 |
| HAT2 |  | 0.663 | 0.361 |  |  | |  |  |  | |  |  |  |
| HAT3 |  | 0.512 | 0.21 |  |  | |  |  |  | |  |  |  |
| HAT4 |  | 0.637 | 0.335 |  |  | |  |  |  | |  |  |  |
|  |  |  |  |  |  | |  |  |  | |  |  |  |
|  |  |  |  |  |  | |  |  |  | |  |  |  |
| **EXPERIMENT 2** | | |  |  |  | |  |  |  | |  |  |  |
|  |  | **OD at 450 nm** | **Sample OD-Blank OD** |  |  | |  |  |  | |  |  |  |
| Blank |  | 0.268 |  |  |  | | **Total histone (µg/µl)** | **OD at 450 nm** | **Sample OD-Blank OD** | | **Slope = 0.010** | **% H4K4ac** | **increase in %H4K4ac** |
| Standard | 100ng/µl | 1.332 | 1.332 |  |  |  |  |  |  |  | **Amount H4K4ac** |  |  |
|  | 50ng/µl | 0.728 | 0.728 |  | Ag83 | | 1.3365 | 0.416 | 0.148 | | 11.074 | 100.00 |  |
|  | 25ng/µl | 0.511 | 0.511 |  | pLPneo2 | | 1.6445 | 0.453 | 0.185 | | 11.250 | 101.59 | 1.588628763 |
|  | 12.5ng/µl | 0.373 | 0.373 |  | HAT1 | | 1.6885 | 0.459 | 0.191 | | 11.312 | 102.15 | 2.150277313 |
|  | 6.25ng/µl | 0.327 | 0.327 |  | HAT2 | | 1.628 | 0.541 | 0.273 | | 16.769 | 151.43 | 51.43124543 |
| Ag83 |  | 0.416 | 0.416 |  | HAT3 | | 1.353 | 0.423 | 0.155 | | 11.456 | 103.45 | 3.452537904 |
| pLPneo2 |  | 0.453 | 0.453 |  | HAT4 | | 1.5015 | 0.499 | 0.231 | | 15.385 | 138.93 | 38.92931393 |
| HAT1 |  | 0.459 | 0.459 |  |  | |  |  |  | |  |  |  |
| HAT2 |  | 0.541 | 0.541 |  |  | |  |  |  | |  |  |  |
| HAT3 |  | 0.423 | 0.423 |  |  | |  |  |  | |  |  |  |
| HAT4 |  | 0.499 | 0.499 |  |  | |  |  |  | |  |  |  |
|  |  |  |  |  |  | |  |  |  | |  |  |  |
|  |  |  |  |  |  | |  |  |  | |  |  |  |
| **EXPERIMENT 3** | | |  |  |  | |  |  |  | |  |  |  |
|  |  | **OD at 450 nm** | **Sample OD-Blank OD** |  |  | |  |  |  | |  |  |  |
| Blank |  | 0.297 |  |  |  | | **Total histone (µg/µl)** | **OD at 450 nm** | **Sample OD-Blank OD** | | **Slope = 0.010** | **% H4K4ac** | **increase in %H4K4ac** |
| Standard | 100ng/µl | 1.273 | 0.976 |  |  |  |  |  |  |  | **Amount H4K4ac** |  |  |
|  | 50ng/µl | 0.859 | 0.562 |  | Ag83 | | 1.3365 | 0.427 | 0.130 | | 9.727 | 100.00 |  |
|  | 25ng/µl | 0.6 | 0.303 |  | pLPneo2 | | 1.6445 | 0.457 | 0.160 | | 9.729 | 100.03 | 0.025726782 |
|  | 12.5ng/µl | 0.475 | 0.178 |  | HAT1 | | 1.6885 | 0.459 | 0.162 | | 9.594 | 98.64 | -1.363066901 |
|  | 6.25ng/µl | 0.418 | 0.121 |  | HAT2 | | 1.628 | 0.541 | 0.244 | | 14.988 | 154.09 | 54.08523909 |
| Ag83 |  | 0.427 | 0.13 |  | HAT3 | | 1.353 | 0.433 | 0.136 | | 10.052 | 103.34 | 3.339587242 |
| pLPneo2 |  | 0.457 | 0.16 |  | HAT4 | | 1.5015 | 0.488 | 0.191 | | 12.721 | 130.78 | 30.77768385 |
| HAT1 |  | 0.459 | 0.162 |  |  | |  |  |  | |  |  |  |
| HAT2 |  | 0.541 | 0.244 |  |  | |  |  |  | |  |  |  |
| HAT3 |  | 0.433 | 0.136 |  |  | |  |  |  | |  |  |  |
| HAT4 |  | 0.488 | 0.191 |  |  | |  |  |  | |  |  |  |
|  |  |  |  |  |  | |  |  |  | |  |  |  |
|  |  |  | **%H4K4 acetylation** | | | | | **Average** |  | |  |  |  |
|  |  |  | **I** | **II** | | **III** | |  |  |  |  |  |  |
|  |  | **WT** | 100.00 | 100.00 | | 100.00 | | 100.00 |  |  |  |  |  |
|  |  | **pLPneo2** | 98.17 | 101.59 | | 100.03 | | 99.93 |  |  |  |  |  |
|  |  | **HAT1** | 99.14 | 102.15 | | 98.64 | | 99.97 |  |  |  |  |  |
|  |  | **HAT2** | 146.71 | 151.43 | | 154.09 | | 150.74 |  |  |  |  |  |
|  |  | **HAT3** | 102.69 | 103.45 | | 103.34 | | 103.16 |  |  |  |  |  |
|  |  | **HAT4** | 147.62 | 138.93 | | 130.78 | | 139.11 |  |  |  |  |  |
|  |  |  |  |  | |  | |  |  |  |  |  |  |
