# Supplementary material for: HAT2 mediates histone H4K4 acetylation and affects micrococcal nuclease sensitivity of chromatin in Leishmania donovani
Source: PLoS One. 2017 May 9;12(5):e0177372. doi: 10.1371/journal.pone.0177372 (PMC5423686; doi:10.1371/journal.pone.0177372)
Supplement: S4 Appendix — (DOC) [file pone.0177372.s008.doc]

**S4** Appendix: Total H3 Acetylation Data

| **EXPERIMENT 1** | | |  |  |  |  | |  |  |  |  |  |  |
| --- | --- | --- | --- | --- | --- | --- | --- | --- | --- | --- | --- | --- | --- |
|  | |  | **Sample RFU** | **Sample RFU- Blank RFU** |  |  | |  |  |  |  |  |  |
| Blank | |  | 49.746 |  |  |  | |  |  |  |  |  |  |
| Standard | | 100ng/µl | 1149.854 | 1100.108 |  |  | | **Qty** | **Sample RFU** | **Sample RFU-Blank RFU** | **Slope = 11.81** | **% H3 acetylation** | **% increase in H3 acetylation** |
|  | | 75ng/µl | 992.577 | 942.831 |  |  |  |  |  |  | **Amount of H3 acetylation** |  |  |
|  | | 50ng/µl | 735.206 | 685.46 |  | Ag83 | | 1.3365 | 85.119 | 35.373 | 2241.058 | 100.00 |  |
|  | | 25ng/µl | 375.869 | 326.123 |  | pLPneo2 | | 1.6445 | 94.869 | 45.123 | 2323.348 | 103.67 | 3.671923685 |
|  | | 6.25ng/µl | 66.515 | 16.769 |  | HAT1 | | 1.6885 | 110.637 | 60.891 | 3053.530 | 136.25 | 36.25395287 |
| Ag83 | |  | 85.119 | 35.373 |  | HAT2 | | 1.628 | 92.227 | 42.481 | 2209.482 | 98.59 | -1.408970883 |
| pLPneo2 | |  | 94.869 | 45.123 |  | HAT3 | | 1.353 | 93.447 | 43.701 | 2734.914 | 122.04 | 22.03675395 |
| HAT1 | |  | 110.637 | 60.891 |  | HAT4 | | 1.5015 | 95.754 | 46.008 | 2594.527 | 115.77 | 15.77241349 |
| HAT2 | |  | 92.227 | 42.481 |  |  | |  |  |  |  |  |  |
| HAT3 | |  | 93.447 | 43.701 |  |  | |  |  |  |  |  |  |
| HAT4 | |  | 95.754 | 46.008 |  |  | |  |  |  |  |  |  |
|  | |  |  |  |  |  | |  |  |  |  |  |  |
| **EXPERIMENT 2** | | |  |  |  |  | |  |  |  |  |  |  |
|  |  | | **Sample RFU** | **Sample RFU-Blank RFU** |  |  | |  |  |  |  |  |  |
| Blank |  | | 47.327 |  |  |  | |  |  |  |  |  |  |
| Standard | 100ng/µl | | 1146.3 | 1098.973 |  |  | | **Qty** | **Sample RFU** | **Sample RFU-Blank RFU** | **Slope = 11.67** | **% H3 acetylation** | **% increase in H3 acetylation** |
|  | 75ng/µl | | 968.7 | 921.373 |  |  |  |  |  |  | **Amount of H3 acetylation** |  |  |
|  | 50ng/µl | | 706.202 | 658.875 |  | Ag83 | | 1.3365 | 91.356 | 44.029 | 2822.923 | 100.00 |  |
|  | 25ng/µl | | 337.569 | 290.242 |  | pLPneo2 | | 1.6445 | 103.719 | 56.392 | 2938.413 | 104.09 | 4.09113908 |
|  | 6.25ng/µl | | 79.325 | 31.998 |  | HAT1 | | 1.6885 | 125.393 | 78.066 | 3961.777 | 140.34 | 40.34308007 |
| Ag83 |  | | 91.356 | 44.029 |  | HAT2 | | 1.628 | 99.628 | 52.301 | 2752.864 | 97.52 | -2.481787188 |
| pLPneo2 |  | | 99.719 | 52.392 |  | HAT3 | | 1.353 | 102.475 | 55.148 | 3492.699 | 123.73 | 23.7263245 |
| HAT1 |  | | 125.393 | 78.066 |  | HAT4 | | 1.5015 | 101.562 | 54.235 | 3095.162 | 109.64 | 9.643893548 |
| HAT2 |  | | 99.628 | 52.301 |  |  | |  |  |  |  |  |  |
| HAT3 |  | | 102.475 | 55.148 |  |  | |  |  |  |  |  |  |
| HAT4 |  | | 101.562 | 54.235 |  |  | |  |  |  |  |  |  |
|  |  | |  |  |  |  | |  |  |  |  |  |  |
|  |  | |  |  |  |  | |  |  |  |  |  |  |
| **EXPERIMENT 3** | | |  |  |  |  | |  |  |  |  |  |  |
|  |  | | **Sample RFU** | **Sample RFU- Blank RFU** |  |  | |  |  |  |  |  |  |
| Blank |  | | 43.885 |  |  |  | |  |  |  |  |  |  |
| Standard | 100ng/µl | | 1073.256 | 1029.371 |  |  | | **Qty** | **Sample RFU** | **Sample RFU-Blank RFU** | **Slope = 11.14** | **% H3 acetylation** | **% increase in H3 acetylation** |
|  | 75ng/µl | | 917.912 | 874.027 |  |  |  |  |  |  | **Amount of H3 acetylation** |  |  |
|  | 50ng/µl | | 699.152 | 655.267 |  | Ag83 | | 1.3365 | 79.983 | 36.098 | 2424.538 | 100.00 |  |
|  | 25ng/µl | | 357.669 | 313.784 |  | pLPneo2 | | 1.6445 | 89.326 | 45.441 | 2480.440 | 102.31 | 2.305698479 |
|  | 12.5ng/µl | | 176.253 | 132.368 |  | HAT1 | | 1.6885 | 107.711 | 63.826 | 3393.215 | 139.95 | 39.95305577 |
| Ag83 |  | | 79.983 | 36.098 |  | HAT2 | | 1.628 | 87.263 | 43.378 | 2391.828 | 98.65 | -1.349123931 |
| pLPneo2 |  | | 88.326 | 44.441 |  | HAT3 | | 1.353 | 90.045 | 46.16 | 3062.547 | 126.31 | 26.31467996 |
| HAT1 |  | | 107.711 | 63.826 |  | HAT4 | | 1.5015 | 89.235 | 45.35 | 2711.233 | 111.82 | 11.82470917 |
| HAT2 |  | | 87.263 | 43.378 |  |  | |  |  |  |  |  |  |
| HAT3 |  | | 90.045 | 46.16 |  |  | |  |  |  |  |  |  |
| HAT4 |  | | 89.235 | 45.35 |  |  | |  |  |  |  |  |  |
|  |  | |  |  |  |  | |  |  |  |  |  |  |
|  |  | |  |  |  |  | |  |  |  |  |  |  |
|  |  | |  |  |  |  | |  |  |  |  |  |  |
|  |  | |  |  |  | **% H3 acetylation** | | | | |  |  |  |
|  |  | |  |  |  | **I** | **II** | | **III** | **Average** |  |  |  |
|  |  | |  |  | WT | 100.00 | 100.00 | | 100.00 | 100.00 |  |  |  |
|  |  | |  |  | pLPneo2 | 103.67 | 104.09 | | 102.31 | 103.36 |  |  |  |
|  |  | |  |  | HAT1 | 136.25 | 140.34 | | 139.95 | 138.85 |  |  |  |
|  |  | |  |  | HAT2 | 98.59 | 97.52 | | 98.65 | 98.25 |  |  |  |
|  |  | |  |  | HAT3 | 122.04 | 123.73 | | 126.31 | 124.03 |  |  |  |
|  |  | |  |  | HAT4 | 115.77 | 109.64 | | 111.82 | 112.41 |  |  |  |
|  |  | |  |  |  |  |  | |  |  |  |  |  |
