# Supplementary material for: HAT2 mediates histone H4K4 acetylation and affects micrococcal nuclease sensitivity of chromatin in Leishmania donovani
Source: PLoS One. 2017 May 9;12(5):e0177372. doi: 10.1371/journal.pone.0177372 (PMC5423686; doi:10.1371/journal.pone.0177372)
Supplement: S5 Appendix — (DOC) [file pone.0177372.s009.doc]

**S5 Appendix: Densitometric quantification of Mononucleosomes and Dinucleosomes from MNase Digested Chromatin**

|  |  | **Quantity** | | | **Average** | **Std. Deviation** |
| --- | --- | --- | --- | --- | --- | --- |
|  |  | **I** | **II** | **III** |
| Spot 1 | *L. donovani* | 39.91539 | 34.47782 | 38.12998 | 37.50773 | 2.771676 |
| Spot 2 |  | 51.83398 | 45.0023 | 46.25941 | 47.69856 | 3.636115 |
| Spot 3 |  | 64.88346 | 60.38443 | 58.11091 | 61.12627 | 3.44668 |
| Spot 4 |  | 100 | 100 | 100 | 100 | 0 |
| Spot 5 | HAT2 over-expressed *L. donovani* | 33.50768 | 35.70365 | 31.52191 | 33.57775 | 2.09175 |
| Spot 6 |  | 48.33583 | 81.10579 | 44.19667 | 57.87943 | 20.22081 |
| Spot 7 |  | 82.93485 | 104.7945 | 81.51553 | 89.74829 | 13.04971 |
| Spot 8 |  | 151.0032 | 155.9794 | 149.0779 | 152.0202 | 3.561389 |
| Spot 9 | *L. donovani* | 44.43596 | 28.06187 | 42.15114 | 38.21632 | 8.867908 |
| Spot 10 |  | 37.41692 | 18.90291 | 34.1864 | 30.16874 | 9.889303 |
| Spot 11 |  | 46.28925 | 23.87771 | 44.00185 | 38.05627 | 12.33214 |
| Spot 12 |  | 70.95942 | 40.54889 | 65.14796 | 58.88542 | 16.14356 |
| Spot 13 | HAT2 over-expressed *L. donovani* | 31.03415 | 33.90829 | 29.42447 | 31.45564 | 2.271431 |
| Spot 14 |  | 45.75512 | 51.95522 | 42.5478 | 46.75271 | 4.782392 |
| Spot 15 |  | 67.66487 | 54.39705 | 61.25231 | 61.10474 | 6.635141 |
| Spot 16 |  | 101.7429 | 86.62479 | 103.5741 | 97.31394 | 9.302243 |

|  | **Mononucleosomes** | |
| --- | --- | --- |
|  | MNase Concentration | Average Quantity |
| *L. donovani Ag83* | 0 U/ml | 37.50773 |
|  | 0.75 U/ml | 47.69856 |
|  | 1.5 U/ml | 61.12627 |
|  | 3.0 U/ml | 100 |
| HAT2 over-expressed *L. donovani* | 0 U/ml | 33.57775 |
|  | 0.75 U/ml | 57.87943 |
|  | 1.5 U/ml | 89.74829 |
|  | 3.0 U/ml | 152.0202 |
|  |  |  |
|  | **Dinucleosomes** | |
|  | MNase Concentration | Quantity |
| *L. donovani Ag83* | 0 U/ml | 38.21632 |
|  | 0.75 U/ml | 30.16874 |
|  | 1.5 U/ml | 38.05627 |
|  | 3.0 U/ml | 58.88542 |
| HAT2 over-expressed *L. donovani* | 0 U/ml | 31.45564 |
|  | 0.75 U/ml | 46.75271 |
|  | 1.5 U/ml | 61.10474 |
|  | 3.0 U/ml | 97.31394 |
